# Mitigating Data Leakage in a WiFi CSI Benchmark for Human Action Recognition

**DOI:** 10.3390/s24248201

**Published:** 2024-12-22

**Authors:** Domonkos Varga

**Affiliations:** Nokia Bell Labs, 1082 Budapest, Hungary; domonkos.varga@nokia-bell-labs.com

**Keywords:** WiFi CSI, human action recognition, machine learning integrity

## Abstract

Human action recognition using WiFi channel state information (CSI) has gained attention due to its non-intrusive nature and potential applications in healthcare, smart environments, and security. However, the reliability of methods developed for CSI-based action recognition is often contingent on the quality of the datasets and evaluation protocols used. In this paper, we uncovered a critical data leakage issue, which arises from improper data partitioning, in a widely used WiFi CSI benchmark dataset. Specifically, the benchmark fails to separate individuals between the training and test sets, leading to inflated performance metrics as models inadvertently learn individual-specific features rather than generalizable action patterns. We analyzed this issue in depth, retrained several benchmarked models using corrected data partitioning methods, and demonstrated a significant drop in accuracy when individuals were properly separated across training and testing. Our findings highlight the importance of rigorous data partitioning in CSI-based action recognition and provide recommendations for mitigating data leakage in future research. This work contributes to the development of more robust and reliable human action recognition systems using WiFi CSI.

## 1. Introduction

Human action recognition using WiFi channel state information (CSI) has gained significant attention due to its non-intrusive nature and potential for applications in areas such as healthcare [1], security [2], and smart homes [3]. Benchmarks and datasets are crucial for evaluating and comparing methods for CSI-based human action recognition. However, recent advancements in this field are often reliant on specific benchmarks, i.e., the WiAR [4] or Widar3.0 databases [5], and the accuracy of these methods can be compromised by inherent flaws in the data.

In this paper, we identify a critical data leakage issue in a widely used WiFi CSI benchmark that results from improper partitioning methods. Specifically, the investigated benchmark published by Moshiri et al. [6] relies heavily on CSI signal-based partitioning, where data from the same individuals are included in both the training and testing sets. This introduces a form of leakage as the models learn specific characteristics of individuals rather than generalizable features of human actions. Ideally, the training and test sets should be partitioned by assigning different individuals entirely to each set, ensuring that the model is evaluated on unseen participants not just on new actions from the same individuals. This form of leakage leads to overly optimistic performance estimates as the models are inadvertently trained on patterns that overlap between the training and testing phases. Consequently, the reported high accuracies do not reflect the model’s ability to generalize to new, unseen subjects—a key requirement for real-world applications of WiFi CSI-based recognition systems.

Our paper provides an in-depth analysis of this issue, quantifies its impact on the several existing models published by Moshiri et al. [6], and proposes strategies to correct the data partitioning method to prevent leakage. We also present revised results using individual-based partitioning, showing a more realistic measure of model performance. By addressing this flaw, we aim to enhance the reliability and robustness of WiFi CSI-based human action recognition research.

This paper offers the following contributions:

1. Identification of the data leakages resulting from the CSI signal-based partitioning in a popular WiFi CSI benchmark.
2. Evaluation of the extent to which this leakage inflates performance metrics.
3. Practical recommendations for preventing data leakage through proper partitioning protocols based on individuals.

### Structure of the Paper

The remainder of this paper is organized as follows. In Section 2, we provide a preliminary overview of WiFi CSI, outlining its relevance and role in human action recognition (HAR). Section 3 reviews related work, focusing on previous approaches of CSI-based HAR and prior studies addressing data leakage issues in machine learning. In Section 4, we describe the materials and methods used and proposed by the authors [6] who developed the benchmark database, highlighting the key features of their approach. Section 5 details the data leakage issue we identified, emphasizing its impact on model performance. Section 6 presents the experimental results, where we retrain the models using both the original partitioning method and a corrected approach that ensures proper partitioning by individuals. Section 7 offers a discussion of our findings, while Section 8 concludes the paper by summarizing our contributions and suggesting future directions for research.

## 2. Preliminaries on WiFi CSI

WiFi CSI refers to a set of parameters that describe the properties of the wireless communication channel between a transmitter and a receiver. It captures the effects of physical factors such as signal attenuation, scattering, reflection, and diffraction as WiFi signals travel through the environment. In essence, CSI provides a detailed snapshot of how the signal interacts with objects and people in the space, offering fine-grained information about signal behavior across different subcarriers and frequency bands. These data, collected from standard WiFi devices, can reveal subtle changes in the environment, making them a powerful tool for applications beyond communication, such as human action recognition.

CSI is derived from the multiple-input multiple-output (MIMO) capabilities of modern WiFi systems, which allow multiple antennas to send and receive data across various transmission paths or channels. When a WiFi signal travels from a transmitter (such as a router) to a receiver (such as a smartphone or laptop), it interacts with various objects and people in its path. These interactions cause variations in the amplitude and phase of the signal. CSI measures these variations at the physical layer, breaking the signal into multiple subcarriers—each corresponding to a different frequency range—and tracking how each subcarrier is affected. The granularity of CSI data provide detailed information about how the signal evolves as it moves through space, capturing environmental dynamics that are not available through simpler metrics like received signal strength indicator (RSSI). Whereas RSSI only measures the overall strength of the received signal, CSI offers a multidimensional view, breaking the signal into multiple subcomponents and tracking how they fluctuate in real time. This makes CSI a rich source of environmental data that can be used to infer changes in the surrounding space. Consider a MIMO system where $N_{t}$ transmit antennas and $N_{r}$ receive antennas are used. Let $H\in C^{N_{r}\times N_{t}}$ represent the channel matrix where each element of H is a complex value that describes the channel between a specific pair of transmit and receive antennas [7]. Formally, the channel matrix can be written as follows:

(1) $$H=\left[\begin{array}{cccc} h_{11} & h_{12} & \cdots & h_{1N_{t}}\\ h_{21} & h_{22} & \cdots & h_{2N_{t}}\\ \vdots & \vdots & \ddots & \vdots \\ h_{N_{r}1} & h_{N_{r}2} & \cdots & h_{N_{r}N_{t}} \end{array}\right],$$

where each element $h_{ij}\in C$ represents the channel response between the *j*th transmit antenna and the *i*th receive antenna. Each $h_{ij}$ is a complex number that models both the amplitude attenuation and phase shift of the signal as it propagates through the channel:

(2) $$h_{ij}=\left|h_{ij}\right|e^{j\theta_{ij}},$$

where $|h_{ij}|$ is the amplitude attenuation of the signal, and $\theta_{ij}$ represents the phase shift introduced by the channel.

The unique properties of CSI make it particularly valuable for HAR [8]. WiFi signals, being ubiquitous and capable of penetrating walls and other obstacles, offer a non-intrusive method to monitor and understand human activities in indoor environments. Unlike cameras [9], which require line-of-sight and can raise privacy concerns, WiFi CSI can be collected passively without direct visual observation [10]. This makes it an appealing solution for applications in healthcare, security, and smart homes, where continuous monitoring of human activity is required.

The core principle behind using CSI for human action recognition is that human movements, such as walking, running, or sitting down, cause measurable disturbances in the WiFi signal [11]. These movements create changes in the signal’s amplitude and phase, which are captured by the CSI. For instance, when a person walks between a WiFi transmitter and receiver, their movement induces multi-path effects, causing the signal to bounce off their body and other surfaces in the environment. These disturbances leave distinct patterns in the CSI data that can be used to identify specific actions. Machine learning algorithms can then be applied to the CSI data to classify and recognize these human actions. By analyzing the variations in the signal, models can be trained to detect a wide range of activities, from simple gestures to more complex movements. For example, researchers have successfully used CSI to differentiate between actions like sitting, standing, walking, and even more granular activities such as hand gestures [12] or breathing patterns [13]. The level of detail provided by CSI allows for high-precision activity recognition, even in challenging environments where traditional sensors or cameras may struggle.

For more detailed information on leveraging WiFi CSI in sensing applications, readers are encouraged to refer to the work of Ma et al. [14], which provides a comprehensive exploration of this topic.

## 3. Related Works

WiFi CSI has emerged as a promising tool for non-intrusive HAR thanks to its ability to capture the fine-grained changes in the wireless channel caused by human movement. Numerous studies have explored the use of CSI for detecting and classifying human activities. In Section 3.1, an overview on the major papers connected to WiFi CSI-based HAR is given. Data leakage occurs when information from the training set is inadvertently included in the test set, leading to overly optimistic performance estimates that fail to generalize to unseen data. In Section 3.2, papers that documented data leakage in various domains of machine learning research are outlined.

### 3.1. WiFi CSI-Based HAR

In the WiSee project, Pu et al. [15] implemented a novel gesture recognition system. Specifically, the WiSee architecture operated by detecting minute Doppler shifts and multi-path distortions in wireless signals caused by human motion. The system was able to classify a set of nine gestures. The proof-of-concept prototype was implemented using USRP-N210 hardware and a software defined radio (SDR) system. Next, it was evaluated in both an office setting and a two-bedroom apartment. Al-qaness’s [16] approach for WiFi CSI-based HAR contained four steps: data collection and normalization, pattern segmentation, feature selection, and activity classification. Specifically, six features were selected both from the CSI amplitude and CSI phase. Based on these features, a random forest [17] algorithm was applied for action classification. WiFall [18] employs anomaly detection algorithms and learns unique CSI patterns to identify falls. It introduces a wireless propagation model designed for indoor environments influenced by human activity and provides a theoretical analysis of the model’s behavior during a fall. E-eyes [19] identifies human activities by analyzing the moving variance of amplitude. This approach is particularly effective for non-stationary activities, especially those with abrupt amplitude changes, like falling and jumping. Conversely, stationary activities, such as sleeping and sitting, show minimal amplitude variation in repetitive patterns, making the moving variance less effective in these cases. The CARM (channel state information-based human activity recognition and monitoring) system [20] incorporates two theoretical models. The first, the CSI speed model, quantifies the relationship between CSI fluctuations and human movement speed, while the second, the CSI activity model, quantifies the connection between human movement speed and specific activities. CDHAR (CSI-based device-free HAR) [21] is a system that uses a WiFi-sensing radar mounted on unmanned aerial vehicles to detect human activities. It employs kernel density estimation to establish adaptive detection thresholds and determine activity duration. For classification, CDHAR utilizes a random subspace ensemble method.

In recent years, deep learning has also been intensively applied to CSI-based HAR. For instance, a long short-term memory (LSTM) network was implemented by Yousefi et al. [7] to recognize human activities using CSI signals. In contrast, Chen et al. [22] implemented an attention-based LSTM for WiFi CSI-based HAR. Similarly, Yang et al. [23] utilized an attention mechanism with LSTM. Schäfer et al. [24] opted to combine the LSTM with a support vector machine for HAR. A deep learning network that integrates hidden features from temporal and spatial dimensions was implemented by Wang et al. [25]. Another line of papers combined convolutional neural network (CNN) and recurrent neural network (RNN) for the spatiotemporal modeling of CSI signals [26,27]. Jiao and Zhang [28] mapped the WiFi CSI-based HAR problem onto the image classification task by converting CSI signals into images using various time series imaging techniques, such as Gramian angular fields [29]. Using the CSI images, a LeNet style [30] CNN was implemented for HAR. This approach was improved by the same authors in [31] by fine tuning on pretrained ImageNet [32] database CNNs. In contrast, Luo et al. [33] fine tuned vision transformers [34] on short-time Fourier transform spectograms [35] that were generated from CSI signals. In [36], Ahmad et al. introduced a state-of-the-art study on the recent advances of WiFi-based HAR and movement tracking using deep learning techniques.

### 3.2. Data Leakage in Machine Learning Research

The growing reliance on machine learning methodologies has revolutionized data analysis across various domains, yet it has also introduced critical challenges, notably data leakage. This phenomenon occurs when information from the test set inadvertently influences the model training process, leading to artificially inflated performance metrics. Such circumstances not only compromise the integrity of research findings, but can also result in misleading conclusions applied in real-world scenarios. As researchers increasingly integrate complex datasets to refine algorithms, understanding the mechanisms and implications of data leakage becomes paramount. Data leakage can lead to overly optimistic performance metrics as models appear proficient in making predictions that they should not have been trained to understand [37]. Essentially, leakage undermines the integrity of model evaluation by providing a false sense of accuracy, complicating the discernment of a models true predictive power. Furthermore, such occurrences may be rooted in improper data preprocessing or inadequate separation of training and validation datasets, thus directly affecting model generalization in real-world applications.

Kaufman et al. [38] discussed the importance of identifying leakage in data mining to ensure accurate predictive modeling. Specifically, the authors stressed the significance of timestamping data to prevent leakage and ensure accurate predictions about the future. In [39], Kapoor and Narayanan delved into the nuances of data leakage within the realm of machine learning-based science. Namely, the authors elaborated a taxonomy of data leakage cases and grouped them into eight classes, i.e., those without a separate test set, preprocessing on the training and test sets, joint feature selection on the training and test sets, duplicated data points, illegitimate features, temporal leakage, non-independence between the training and test sets, and sampling bias. The authors underscored that the training dataset must be clearly separated from the test dataset during preprocessing, modeling, and evaluation steps. Further, a machine learning model should have no access to illegitimate features. For instance, Filho et al. [40] pointed out that Ye et al. [41] applied anti-hypertensive drugs as a feature in a hypertension prediction task. As a result, data leakage was introduced since the model would not have this information during the prediction phase.

## 4. Materials and Methods

Moshiri et al. [6] collected CSI data using a Raspberry Pi device equipped with a network interface card. Specifically, the authors collected CSI data for seven different human activities, i.e., fall, stand up, sit down, lie down, run, walk, and bend. Each activity was performed by three different users twenty times. For more details on the experimental setup, we refer readers to the 5.1 Subsection of Moshiri et al.’s [6] journal paper. The authors made publicly available the collected database, which is referred as CSI-HAR database in the following: the CSI-HAR database is available at: https://github.com/parisafm/CSI-HAR-Dataset accessed on 19 December 2024. Consequently, the dataset created for the study of [6] is publicly available and consists of 420 samples collected from three volunteers performing the specified activities. The authors plan to expand this dataset and explore various scenarios, including interactions between multiple users and activities performed by individuals of different ages.

**Figure 1:**
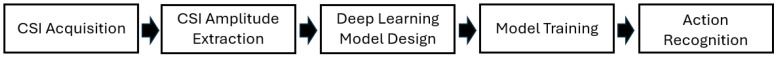
Generalized deep learning framework applied by Moshiri et al. [6] for WiFi CSI-based human action recognition.

**Figure 2:**
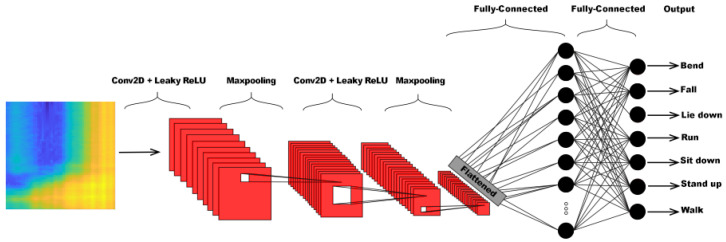
Structure of the 2D-CNN for the WiFi CSI-based HAR proposed by Moshiri et al. [6].

**Figure 3:**
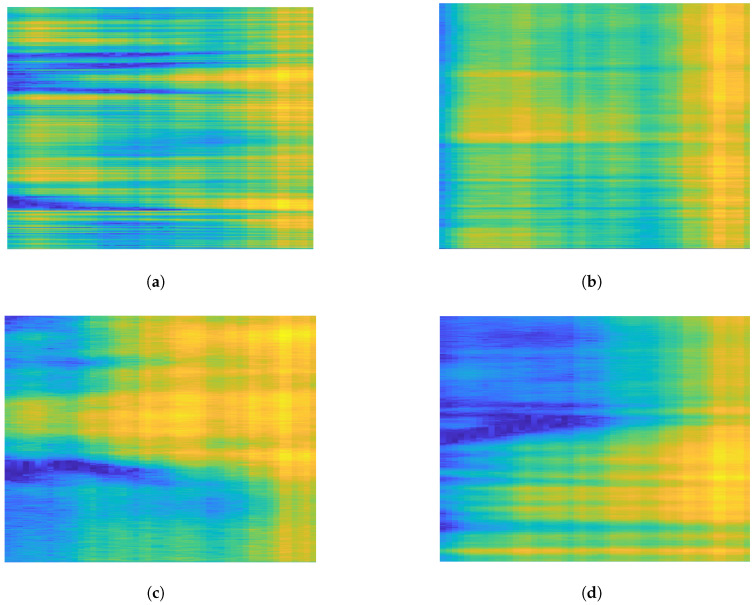
Illustration of CSI images of various human actions in the CSI-HAR database [6]. The CSI signals (52 channels in CSI-HAR [6]) were converted to RGB images using MATLAB 2022B’s imagesc function [43]. (**a**) Walk. (**b**) Fall. (**c**) Stand up. (**d**) Sit down.

**Figure 4:**
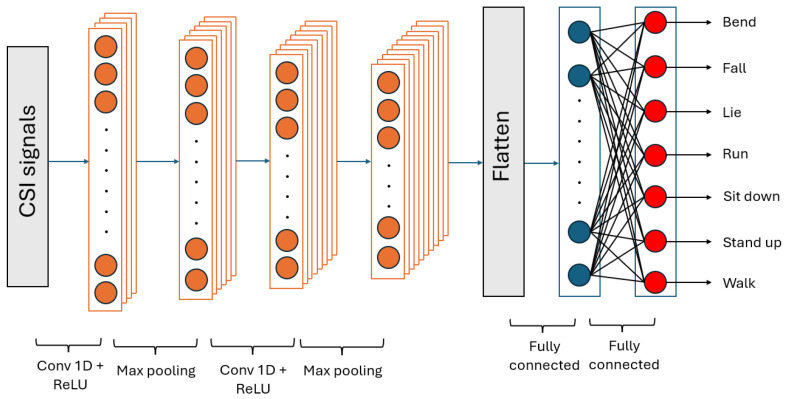
Structure of the 1D-CNN for the WiFi CSI-based HAR proposed by Moshiri et al. [6].

The generalized framework of Moshiri et al.’s [6] proposed methods is depicted in Figure 1. As one can see, the WiFi CSI-based HAR is formulated as a classification task, and various deep learning algorithms were designed for HAR in [6]. The authors proposed four different deep learning architectures to solve the task: 2D-CNN, 1D-CNN, LSTM, and BLSTM (bi-directional long short-term memory). The structure of the 2D-CNN is depicted in Figure 2. Following the first convolutional layer, which uses a leaky ReLU activation function and max pooling, batch normalization (BN) [42] is introduced to help stabilize and accelerate network training. BN stabilizes the estimation of mean and standard deviation across mini-batches, bringing them closer to values of 0 and 1, respectively. Dropout layers are incorporated between convolutional layers to reduce overfitting and enhance the network’s generalization ability. The pooled features, obtained from max pooling, need to be flattened by transforming the feature map matrix into a single-column matrix. This flattened matrix is then fed through two fully connected layers to obtain the predicted class outputs. Unlike 1D-CNN, LSTM, and BLSTM, 2D-CNN was not trained on one-dimensional CSI signals but the corresponding CSI signals (52 channels in the CSI-HAR [6] database) were converted to RGB images using MATLAB’s imagesc [43] function. Illustration of the CSI images of various human actions can be seen in Figure 3. The structure of the 1D-CNN, which is very similar to those of 2D-CNN, can be seen in Figure 4.

**Figure 5:**
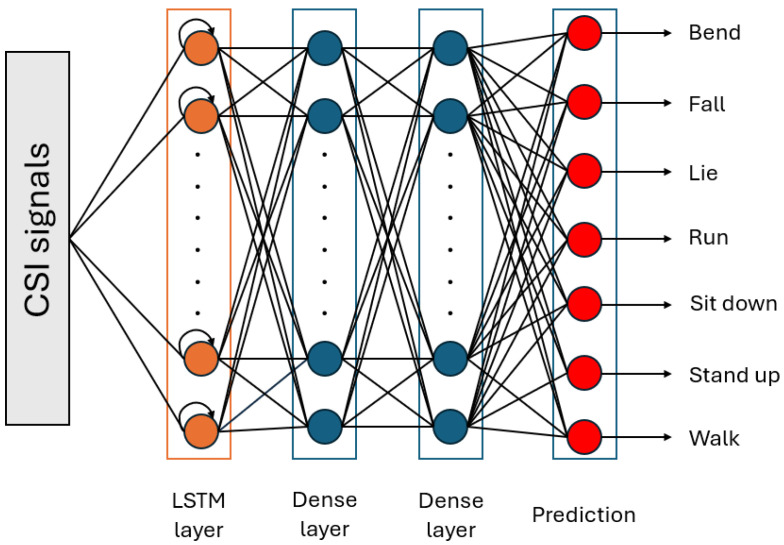
Structure of the LSTM for the WiFi CSI-based HAR proposed by Moshiri et al. [6].

Due to the time-series nature of CSI signals and the LSTM’s [44] capability to capture complex temporal dynamics, Moshiri et al. [6] expected that an LSTM network would demonstrate an outstanding performance in WiFi CSI-based HAR. For the HAR task, LSTM offers two key benefits: it can automatically extract features without needing preprocessing; and it retains the temporal state information of activities, leading to better performance for similar actions, like lying down versus sitting down, compared to 1D-CNN or the hidden Markov model. In [6], the authors used a simple LSTM with one hidden layer containing 128 hidden units, where the feature vector consists of a 52-dimensional vector of CSI amplitudes. The proposed LSTM architecture is shown in Figure 5.

**Figure 6:**
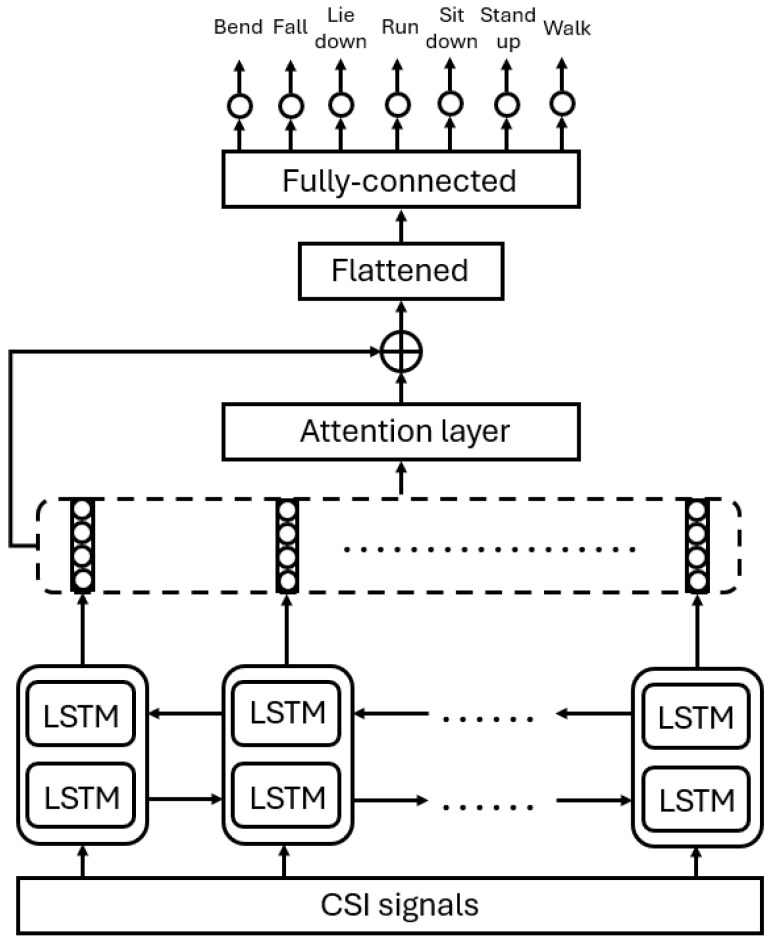
Structure of the BLSTM for the WiFi CSI-based HAR proposed by Moshiri et al. [6].

The conventional LSTM network analyzes CSI data in a single direction, meaning the current hidden state only takes into account past CSI information. However, future CSI data are also crucial for HAR. This is why, Moshiri et al. [6] made experiments with an attention-based BLSTM to capture both past and future information, addressing long-term dependencies. Specifically, the network of Moshiri et al. [6] includes both forward and backward layers, enabling it to extract information from both directions. Essentially, it is a two-layer LSTM model where one processes the input in a forward direction and the other in reverse. Attention [45], as the name suggests, is a method that enables the model to focus on specific timesteps in input sequences of arbitrary length. Given that the sequential features learned by the BLSTM network for WiFi-based HAR are high-dimensional, with varying feature contributions and time steps depending on the case, the attention model was leveraged to automatically determine the significance of features and to adjust their weights based on recognition performance. In the study of Moshiri et al. [6], as shown in Figure 6, a BLSTM with an attention layer consisting of 400 units was employed to learn the relative importance of features and timesteps, giving higher weights to more important features to improve performance.

The parameters of the training, which were applied in the training process of 2D-CNN, 1D-CNN, LSTM, and BLSTM, are shown in Table 1. The methods proposed by Moshiri et al. [6] were reimplemented using Python 3.11.5 and PyTorch 2.5.1 in a computer configuration, as given in Table 2.

## 5. Detected Data Leakage

**Figure 7:**
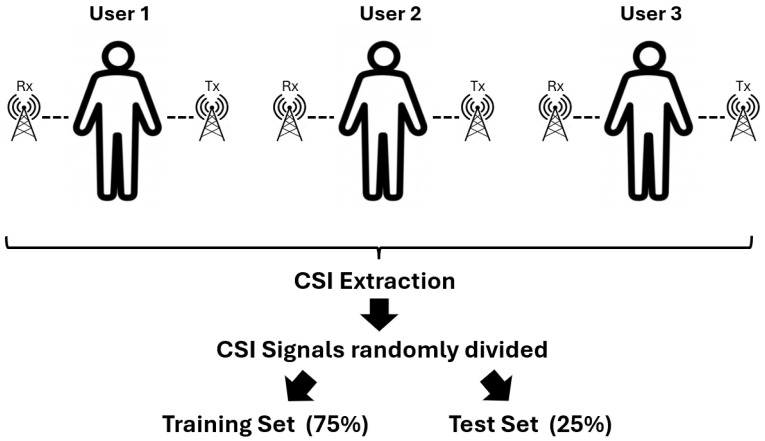
Data split without respect to humans. This figure illustrates the improper data partitioning method applied by Moshiri et al. [6], where data from the same individuals are included in both training and test sets. Specifically, the CSI signals are extracted first and then randomly allocated to a training set (75% of signals) and a test set (25% of signals). As a result, a machine or deep learning model learns individual-specific features rather than general patterns of human actions, leading to data leakage and inflated performance metrics.

**Figure 8:**
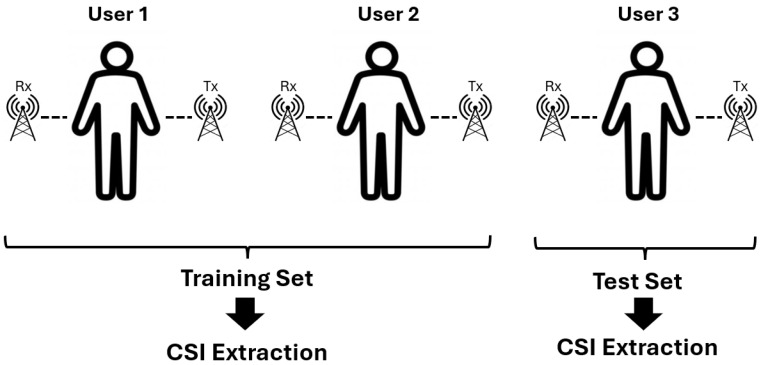
Data split with respect to humans. This figure illustrates the correct data partitioning method, where different individuals are assigned entirely to the training and test sets. Next, CSI signal extraction was carried out. This approach ensures that the model is evaluated on unseen individuals, preventing data leakage and promoting generalization of human action recognition models.

When data are split without considering individual separation, the training and test sets both include data from the same individuals. This approach can lead to significant data leakage as the model may inadvertently learn person-specific features rather than generalized action patterns. As shown in Figure 7, the improper data split—which was applied by Moshiri et al. [6]—allows the model to memorize unique attributes associated with individuals, leading to inflated performance metrics that do not reflect the model’s true generalization ability. To address this issue, we introduced a data split with respect to individuals, as shown in Figure 8, where distinct sets of individuals are assigned to either the training or the test set but not both. This approach ensures that the model is evaluated on previously unseen individuals, reducing the risk of data leakage and promoting the learning of action-specific features that are more generalizable across different individuals. By isolating the training and test sets based on individuals, the model is forced to focus on patterns relevant to actions rather than individual-specific characteristics. In our experiments, the data from the first two volunteers of CSI-HAR [6] were allocated to the training set, and the data of the third volunteer were allocated to the test set. Our experiments revealed that this corrected partitioning method significantly impacts the model’s accuracy, highlighting the importance of rigorous data splitting strategies in WiFi CSI-based human action recognition tasks. The findings underscore the need for careful evaluation protocols to prevent data leakage and support the development of robust and generalizable HAR systems.

Data partitioning with respect to individuals is not only critical for evaluating model performance accurately in research, but it is also essential in the context of production development. In real-world applications of WiFi CSI-based human action recognition—such as healthcare monitoring, security, and smart home environments—models must generalize well to new users who were not part of the training data. If a model’s performance is assessed using the same individuals in both the training and test sets, its reported accuracy is likely overstated, reflecting its ability to memorize specific individual characteristics rather than recognizing generalized human actions. Without individual-based partitioning, models risk failing in real production environments where they will encounter previously unseen individuals. This misalignment between research results and real-world performance can lead to unreliable systems that struggle to maintain accuracy when deployed at scale. For example, in healthcare settings where accurate monitoring of activities is crucial, a model that cannot generalize across individuals may miss critical events or generate false positives, potentially compromising patient safety and the system’s trustworthiness. By enforcing individual-based data splits during development, we simulated real-world deployment conditions more accurately, encouraging the model to learn robust features that are independent of any particular user’s characteristics. This approach promotes the creation of generalizable models that are better equipped for large-scale deployment, where diverse populations and varied environments are the norm. In turn, this leads to more reliable and consistent performance, enhancing the model’s utility and reliability in production settings.

## 6. Results

### 6.1. Evaluation Metrics

To ensure the validity and comparability of our findings, we adopted the exact evaluation metrics used by Moshiri et al. in their paper [6]. This approach not only verified our results against the original benchmarks, but it also strengthened the credibility of our analysis of data leakage and its impact on model reliability. In machine learning-based classification, accuracy is a widely used evaluation metric that measures the proportion of correct predictions made by a model over the total number of predictions. Formally, the accuracy for multi-class classification can be defined as follows:

(3) $$Accuracy=\frac{\mathrm{Number}\;\mathrm{of}\;\mathrm{Correct}\;\mathrm{Predictions}}{\mathrm{Total}\;\mathrm{Number}\;\mathrm{of}\;\mathrm{Predictions}}=\frac{\sum_{i=1}^{C}TP_{i}}{\sum_{i=1}^{C}\left(TP_{i}+FP_{i}+FN_{i}\right)},$$

where

- *C* represents the total number of classes;
- $TP_{i}$ denotes the true positives for class *i*;
- $FP_{i}$ and $FN_{i}$ represent the false positives and false negatives for class *i*, respectively.

In the context of classification tasks, accuracy provides a measure of the model’s overall effectiveness by indicating the percentage of correct predictions across all classes. However, it is worth noting that while accuracy is a straightforward metric, it may not fully reflect the model performance in cases of imbalanced data, where one class is significantly more frequent than others. In such scenarios, additional metrics such as precision, recall, and F1 score can offer more insight into a model’s performance across different classes. However, CSI-HAR [6] is balanced with an equal distribution across all classes. As a result, accuracy serves as an appropriate and sufficient evaluation metric, providing a clear and reliable measure of model performance without the need for additional metrics to account for class imbalance.

### 6.2. Numerical Results

The numerical results of Moshiri et al.’s [6] methods using the two different data partitioning strategies are illustrated in Table 3. From these results, it can be seen that we could only replicate the published results when employing a data split that did not take individuals into account. In the case of the correct data split (with respect to humans), the results declined to approximately 63% to 72% of the published performance metrics. This substantial reduction underscores the impact of proper individual-based partitioning on model evaluation, revealing that the initially reported results may have been inflated due to data leakage.

**Figure 9:**
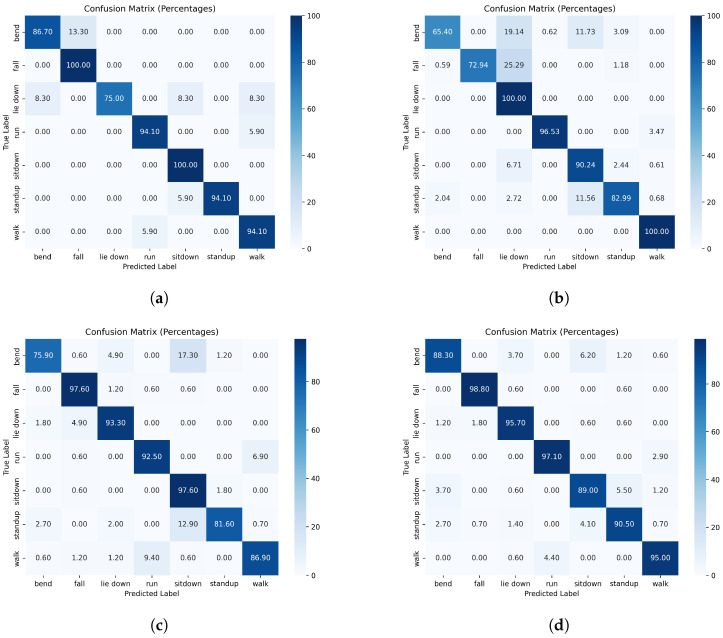
Confusion matrices of Moshiri et al.’s [6] methods if the CSI data are split without respect to humans: (**a**) 2D-CNN; (**b**) 1D-CNN; (**c**) LSTM; and (**d**) BLSTM.

**Figure 10:**
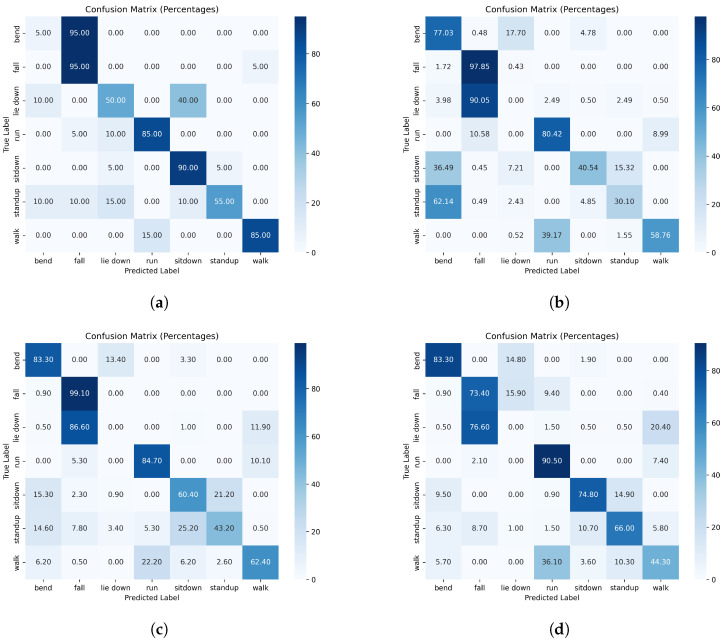
Confusion matrices of Moshiri et al.’s [6] methods if the CSI data are split with respect to humans: (**a**) 2D-CNN; (**b**) 1D-CNN; (**c**) LSTM; and (**d**) BLSTM.

In Figure 9 and Figure 10, the confusion matrices obtained with data split without and with respect to humans are depicted, respectively. A confusion matrix in multi-class classification with percentages showed the proportions of actual versus predicted class labels, where each cell represents the percentage of instances relative to the total, with correct predictions on the diagonal and misclassifications on the off-diagonal [47]. In the case of data splits without respect to individuals, the confusion matrices showed uniformly high values along the diagonal, indicating that the networks achieved consistently high accuracy across all action types. This uniformity suggests that no specific action type posed a significant challenge, likely due to the networks leveraging individual-specific features rather than action-specific patterns. In the case of the individual-respecting data split, the confusion matrices revealed distinct challenging cases. For example, the “lie down” action showed notably low accuracy, indicating that this action is more difficult for the network to identify accurately. In contrast, the “fall” action maintained high accuracy, similar to the results in the without-individual-respecting case, suggesting that it remains an easily recognizable action for the network.

**Figure 11:**
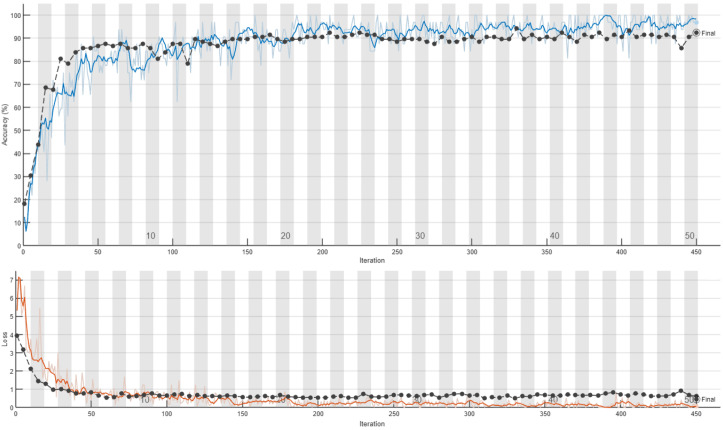
Retraining of 2D-CNN on the CSI-HAR database [6] without respect to humans. In the upper section of the figure, the training accuracy is represented by a blue line and the test accuracy by a black line. In the lower section of the figure, the red line indicates training loss, while the black line shows the loss on the test set.

**Figure 12:**
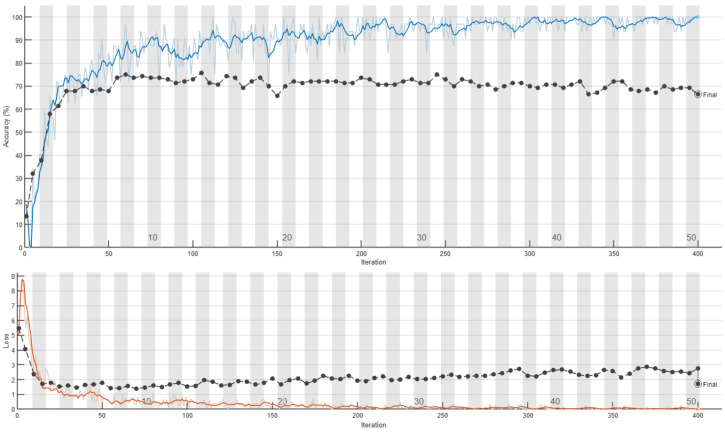
Retraining of 2D-CNN on the CSI-HAR database [6] with respect to humans. In the upper section of the figure, the training accuracy is represented by a blue line and the test accuracy by a black line. In the lower section of the figure, the red line indicates training loss, while the black line shows the loss on the test set.

The training curves of the 2D-CNN model with data splits without and with respect to individuals are depicted in Figure 11 and Figure 12, respectively. These figures illustrate the differences in the model training behavior under each partitioning strategy, highlighting the impact of individual-based separation on the network’s learning process and final performance. Several interesting conclusions can be drawn from these two figures. When the data are split without regard for individual subjects, a close alignment between training and test accuracies can be observed, with test accuracy following the training trend closely and showing only minor differences. In contrast, when the data split respects individual subjects, a marked divergence arises between training and test accuracy. Although training accuracy continued to improve steadily, the test accuracy quickly reached a plateau, indicating that the model struggles to generalize to new, unseen data. This gap suggests that the model may be overfitting to the training data under subject-based partitioning, underscoring the importance of sound data management practices to ensure model robustness and generalizability. The striking contrast between training and test accuracy behavior highlights how crucial the choice of data partitioning strategy is for accurately assessing model performance. These findings emphasize the need for subject-based partitioning to achieve reliable estimates of model generalization and to reduce the risk of overfitting.

## 7. Discussion

Our analysis highlights the substantial impact of data partitioning strategies on the performance of the WiFi CSI-based human action recognition models introduced and published in [6]. In our experiments, when individuals were not separated between the training and test sets, the models achieved accuracy levels very close to those reported in the original publication. However, these results were largely influenced by data leakage as the networks were able to leverage individual-specific features, leading to inflated performance metrics. The confusion matrices for this case showed consistently high values along the diagonal, indicating uniformly strong performance across action types and suggesting that no particular actions were overly challenging for the models. This finding underscores the misleading nature of evaluations that do not account for individual-based partitioning as they do not accurately reflect the models’ generalizability to new users.

Conversely, when the correct data split (with respect to individuals) is applied, the models’ performance declined significantly, achieving only 63% to 72% of the original reported accuracy. This drop illustrates the importance of rigorously isolating individuals in training and testing to ensure fair evaluation. The confusion matrices for the individual-respecting split provide further insight, showing that certain actions, such as “lie down”, were particularly challenging for the models, resulting in a much lower accuracy. On the other hand, actions like “fall” maintained high accuracy, similar to the results seen in the original, non-individual-respecting split, which was likely due to the distinct and robust patterns associated with this action.

These findings underscore the need for more stringent evaluation practices in CSI-based human action recognition. Models developed without proper data partitioning risk poor performance when deployed in real-world environments, where they encounter previously unseen individuals [48]. Our results demonstrate that partitioning data with respect to individuals promotes the learning of action-specific rather than individual-specific features, enhancing the model’s potential for real-world application.

In light of these observations, we recommend that future studies prioritize rigorous data splitting protocols to avoid data leakage and ensure generalizable results. Additionally, using supplementary evaluation metrics beyond accuracy, such as per-class precision and recall, could provide deeper insight into the model’s strengths and limitations for each action class. Overall, this work contributes to establishing best practices in CSI-based human action recognition and highlights the importance of robust evaluation to advance the field toward reliable and deployable solutions.

## 8. Conclusions

In this work, we examined the critical impact of data partitioning strategies on the performance of WiFi CSI-based human action recognition models. Our investigation revealed that data leakage occurs when the same individuals are included in both training and test sets, leading to inflated accuracy metrics due to the model’s reliance on individual-specific features rather than generalized patterns of human actions. When applying an individual-respecting split, the model’s performance dropped to 63–72% of the originally reported metrics, underscoring the importance of rigorous evaluation protocols. These findings emphasize the necessity of proper data partitioning to ensure reliable model performance and generalizability, particularly for real-world applications, where models are expected to handle new, unseen individuals. By highlighting the significant discrepancy in performance caused by data leakage, our work encourages the adoption of best practices in CSI-based human action recognition, particularly regarding data management and model evaluation. Future research should continue to prioritize robust validation strategies to advance the field toward creating deployable, reliable systems that maintain high accuracy in practical scenarios. 

## Figures and Tables

**Table 1:** Parameter settings.

Parameter	Value
Loss function	Cross-entropy
Optimizer	Adam [46] $\left(\beta_{1}=0.9,\beta_{2}=0.99,\epsilon_{2}=1\times 10^{-9}\right)$
Learning rate	0.001
Decay rate	0.8
Batch size	64
Epochs	50

**Table 2:** Computer configurations.

Computer model	STRIX Z270H Gaming
Operating system	Windows
CPU	Intel(R) Core(TM) i7-7700K CPU 4.20 GHz (8 cores)
Memory	15 GB
GPU	NVIDIA GeForce GTX 1080

**Table 3:** Comparison of the results on CSI-HAR [6].

	Reported in [6]	Retrained w/o.r.t. Humans	Retrained w.r.t. Humans
Architecture	Accuracy	Accuracy	Accuracy
2D-CNN	95.5%	92.2%	66.4%
1D-CNN	87.4%	86.9%	55.0%
LSTM	89.2%	89.3%	61.8%
BLSTM	94.7%	93.6%	62.2%

## Data Availability

The data used in this study were derived from the CSI-HAR database available for download at https://github.com/parisafm/CSI-HAR-Dataset (accessed on 19 December 2024). For this study, the data were rearranged to address specific research questions, and the rearranged dataset is available for download at https://github.com/elektrische-schafen/CSI-HAR-Database (accessed on 19 December 2024).

## Abbreviations

The following abbreviations are used in this manuscript:

BLSTM	bi-directional long short-term memory
BN	batch normalization
CNN	convolutional neural network
CPU	central processing unit
CSI	channel state information
FN	false negative
FP	false positive
GPU	graphics processing unit
MIMO	multiple-input multiple-output
HAR	human action recognition
LSTM	long short-term memory
RNN	recurrent neural network
RSSI	received signal strength indicator
SDR	software defined radio
TP	true positive
